# Use of Human Cancer Cell Lines Mitochondria to Explore the Mechanisms of BH3 Peptides and ABT-737-Induced Mitochondrial Membrane Permeabilization

**DOI:** 10.1371/journal.pone.0009924

**Published:** 2010-03-31

**Authors:** Nelly Buron, Mathieu Porceddu, Magali Brabant, Diana Desgué, Cindy Racoeur, Myriam Lassalle, Christine Péchoux, Pierre Rustin, Etienne Jacotot, Annie Borgne-Sanchez

**Affiliations:** 1 THERAPTOSIS S.A., Oncology Department, Biocitech Technology Park, Romainville, France; 2 MITOLOGICS S.A.S., Mitologics Research Laboratory, Hôpital Robert Debré, Paris, France; 3 INRA, UR1196 Génomique et Physiologie de la Lactation, Plateau de Microscopie Électronique MIMA2, Jouy-en-Josas, France; 4 Inserm U676, Hôpital Robert Debré, Paris, France; 5 Université Paris 7, Faculté de Médecine Denis Diderot, Paris, France; 6 Imperial College London, Department of Reproductive Biology, Cancer Division, Hammersmith Hospital, London, United Kingdom; Health Canada, Canada

## Abstract

Current limitations of chemotherapy include toxicity on healthy tissues and multidrug resistance of malignant cells. A number of recent anti-cancer strategies aim at targeting the mitochondrial apoptotic machinery to induce tumor cell death. In this study, we set up protocols to purify functional mitochondria from various human cell lines to analyze the effect of peptidic and xenobiotic compounds described to harbour either Bcl-2 inhibition properties or toxic effects related to mitochondria. Mitochondrial inner and outer membrane permeabilization were systematically investigated in cancer cell mitochondria *versus* non-cancerous mitochondria. The truncated (t-) Bid protein, synthetic BH3 peptides from Bim and Bak, and the small molecule ABT-737 induced a tumor-specific and OMP-restricted mitochondrio-toxicity, while compounds like HA-14.1, YC-137, Chelerythrine, Gossypol, TW-37 or EM20-25 did not. We found that ABT-737 can induce the Bax-dependent release of apoptotic proteins (cytochrome c, Smac/Diablo and Omi/HtrA2 but not AIF) from various but not all cancer cell mitochondria. Furthermore, ABT-737 addition to isolated cancer cell mitochondria induced oligomerization of Bax and/or Bak monomers already inserted in the mitochondrial membrane. Finally immunoprecipatations indicated that ABT-737 induces Bax, Bak and Bim desequestration from Bcl-2 and Bcl-xL but not from Mcl-1L. This study investigates for the first time the mechanism of action of ABT-737 as a single agent on isolated cancer cell mitochondria. Hence, this method based on MOMP (mitochondrial outer membrane permeabilization) is an interesting screening tool, tailored for identifying Bcl-2 antagonists with selective toxicity profile against cancer cell mitochondria but devoid of toxicity against healthy mitochondria.

## Introduction

Apoptosis dysregulation has been shown to underly several pathologies including cancer [1], [2]. It is well established that diverse signalling events within apoptosis converge on mitochondria which undergo outer membrane permeabilization (OMP) triggering the release of soluble apoptogenic factors from the intermembrane space such as cytochrome c and a subsequent series of activation of a set of proteolytic enzymes, the caspases conducting to apoptotic dismantling of cell structure [3].

MOMP is under the control of members of the Bcl-2 protein family which includes (1) anti-apoptotic proteins like Bcl-2, Bcl-xL, Bcl-w, Mcl-1 and A1/Bfl-1 containing all four Bcl-2 homology domains (BH1-4), (2) pro-apoptotic proteins like Bax, Bak, Bok lacking the BH4 domain and (3) pro-apoptotic BH3-only proteins like Bid, Bim, Bad, Bmf, Noxa and Puma [4]–[8]. In the direct activation model, induction of Bim or Bid is required for Bax or Bak to oligomerize and form pores in the outer mitochondrial membrane (MOM) [9], [10]. The anti-apoptotic proteins can block this process at the MOM by primarily sequestering Bax/Bak proteins [11]–[13]. In the indirect activation model [14], [15], BH3-only proteins can antagonize anti-apoptotic effect and liberate Bax/Bak proteins. It is still a matter of debate whether Bax and Bak may interact with proteins like VDAC (voltage dependent anion channel) and/or ANT (adenine nucleotide translocator) to regulate the permeability transition pore (PTP) [16]. At the mitochondrial level, the cytochrome c is distributed in two distinct pools: 15–20% in the intermembrane space and the larger fraction (80%) in the intracristae space [17]. Thus, BH3 mimetic peptide needs matrix remodeling to release the second pool of cytochrome c [18]. Other apoptotic factors like Omi/HtrA2 and Smac/DIABLO (caspase-dependent death effectors) or the apoptosis-inducing factor AIF and EndoG (caspase-independent death effectors) are released after MOMP.

The mitochondrial membrane permeabilization (MMP) process is often altered in cancer cells possibly as a result of PTP component overexpression [19], upregulation of anti-apoptotic members of the Bcl-2 family and/or downregulation of Bax [20]. These underly numerous anti-cancer strategies targeting components of the core cell death machinery to promote tumor cell death [21], [22]. These strategies are based on the use of BH3-mimicking peptides [14], [23], antisense [24] or RNA interference [25] against Bcl-2, and natural or synthetic small molecules which bind specifically to Bcl-2 family proteins. For instance screening approaches using nuclear magnetic resonance, structure-based design and combinatory chemical synthesis, led to the identification of ABT-737, a small-molecule inhibitor of the anti-apoptotic proteins Bcl-2, Bcl-xL and Bcl-w but not Mcl-1 and A1/Bfl1 [26]. ABT-737 is considered to be a Bad-like BH3 mimetic because both ABT-737 and Bad BH3 peptide bind the same subset of Bcl-2 pro-survival proteins [27] and induce cytochrome c release in mitochondria obtained from “primed for death” tumor cells [28]. However, the weak affinity of ABT-737 for the pro-survival proteins Mcl-1 and A1/Bfl1 [26] might be a key determinant of tumor cell resistance to this compound [29].

We have set up a multiparametric screen on purified mitochondria to identify compounds inducing OMP of mitochondria isolated from cancer cell lines, but not of mitochondria isolated from non-cancerous cells. Among various compounds (from chemical, peptidic or proteic origins) described to target mitochondria, we found that only recombinant t-Bid, Bak BH3 and Bim BH3 peptides, and ABT-737 present a direct tumor-specific mitochondrio-toxicity and induce relatively large OMP due to Bax and Bak oligomerization. By further exploration of ABT-737-induced OMP at the cell-free mitochondrial level, we found that (1) cancer cell mitochondria from different sources differed in their sensitivity to ABT-737 correlating with different patterns of (outer) membrane-associated Bcl-2 family members and their interactions, (2) ABT-737 induces Bax, Bak, and Bim desequestration from Bcl-xL and Bcl-2, but not from Bcl-w or Mcl-1.

## Results

### Isolation and functional characterization of healthy and tumor mitochondria

**Figure 1:**
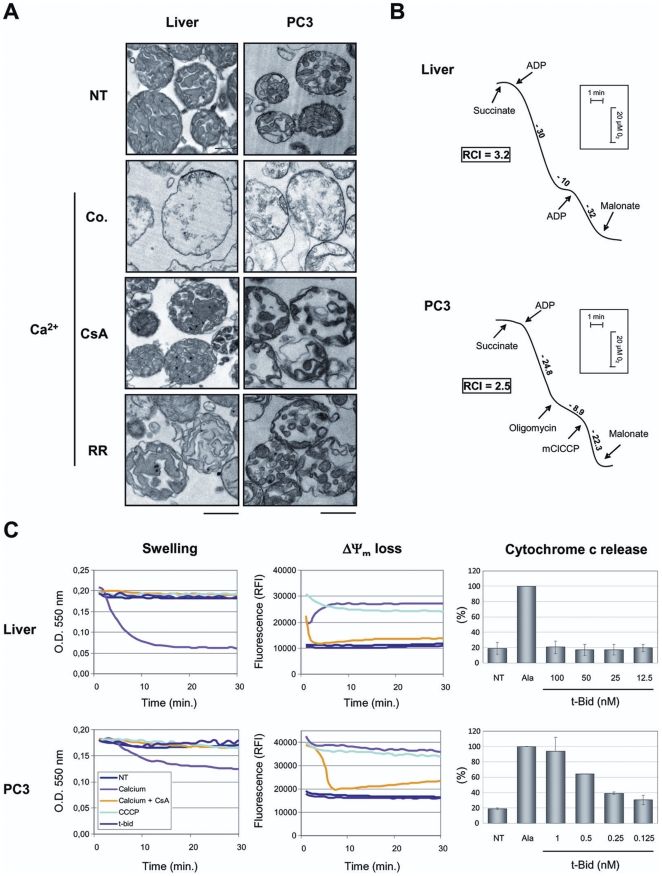
Isolation and functional characterization of mouse liver and human tumor cell line mitochondria. **A.** Ultrastructural analysis of isolated mitochondria and their ability to swell. Electron micrographs were obtained after incubation of mitochondria isolated from healthy mouse liver, or PC-3 tumor cell lines untreated (NT) or treated with Ca^2+^ (50 µM) or with a 5 min-preincubation with cyclosporin A (CsA; 10 µM) or ruthenium red (RR; 1 µM) before calcium addition. The percentage of swollen mitochondria was <10% in the control and >80% 30 min after Ca^2+^ addition (n = 3). Scale bars 1 µm. **B.** Oxidative properties of isolated liver and PC-3 mitochondria. Traces represent oxygen consumption by isolated mitochondria (100 µg) after addition of the indicated reagents. Numbers along the trace are nmoles of O_2_ consumed per minute per milligram of protein. The respiratory control index (RCI) is calculated for each type of mitochondria as indicated in Materials and Methods. **C.** To evaluate mitochondrial swelling and ΔΨ_m_ loss, mitochondria isolated from healthy mice liver or PC-3 cell line were distributed in 96-well microplates and incubated for 30 min either with Ca^2+^ (100 µM) in presence (yellow) or absence (pink) of CsA (10 µM), with *m*ClCCP (turquoise; 50 µM) or with t-Bid (purple; 1 nM). For quantitation of cytochrome c release, isolated mitochondria were treated with increasing concentrations of the t-Bid recombinant protein and mitochondrial supernatant was subjected to ELISA assays, given in percentage of release compared to 20 µg/ml alamethicin (Ala; 100% of cytochrome c release) (n = 3 independent experiments).

Mitochondria from both healthy tissue (mouse liver) and human tumor (PC-3, prostate) cell line were purified by isopycnic centrifugation in density gradients of Percoll. The isolated mitochondria were found highly intact as demonstrated by cytochrome c oxidase accessibility assay and flow cytometry FSC/SSC analysis [30]. Ultrastructural comparative studies of isolated mitochondria from liver or PC-3 tumor cell line reveal a relatively similar matrix/cristae organization despite a slight difference in density between tumor (low density) and liver (high density) mitochondria (Fig. 1A). Calcium (50 µM) induces an extensive outer membrane disruption in both healthy tissue and tumor cell line mitochondria followed by a swelling which is inhibited by cyclosporine A (CsA), indicating an intact and functional permeability transition pore in both mitochondrial types. Polarographic investigations were next carried out on liver and PC-3 mitochondria (Fig. 1B). Succinate oxidation was essentially dependent on ADP addition and a respiratory control index (RCI) of 3 associated with succinate oxidation indicated the functional integrity of mitochondria, including those isolated from tumor cultured cells. Similarly, mitochondria isolated from HT-29, HCT-116 and Jurkat cancer cell lines and HME-1 non-cancerous cell line presented high level of integrity and functionality (not shown).

### Multiparametric screening method on isolated healthy and tumor mitochondria

Isolated mitochondria were analyzed on a screening platform which allowed the quantification of the mitochondrial membrane permeabilization (swelling; Fig. 1C; left panel) plus mitochondrial transmembrane potential (ΔΨm; Fig. 1C, middle panel) using real-time spectrofluorimetry and cytochrome c release by ELISA as an index for MOMP (Fig. 1C; right panel). Real-time ΔΨm detection reflected inner membrane and respiratory chain alterations but did not permit to observe delayed ΔΨm in response to pro-apoptotic compounds. When incubated in hypotonic buffers, both normal and tumoral cell mitochondria did swell (loss of O.D. at 550 nm) in the presence of calcium in a CsA-dependent manner. However, the swelling amplitude was reduced in the case of tumor mitochondria in agreement with their lowest density compared to liver mitochondria. Calcium and *m*ClCCP induced a rapid ΔΨm loss characterized by an increased fluorescence corresponding to Rhodamine-123 dequenching due to a decrease of the dye's concentration in depolarized mitochondria. We thus observed that the recombinant protein t-Bid had no effect on swelling and ΔΨm but induced cytochrome c release specifically in PC-3 (Fig. 1C), HT-29, HCT-116 and Jurkat (not shown) cell mitochondria in a concentration-dependent manner as indicated by ELISA analysis of the supernatants.

### Screening of putative Bcl-2 family inhibitors

**Figure 2:**
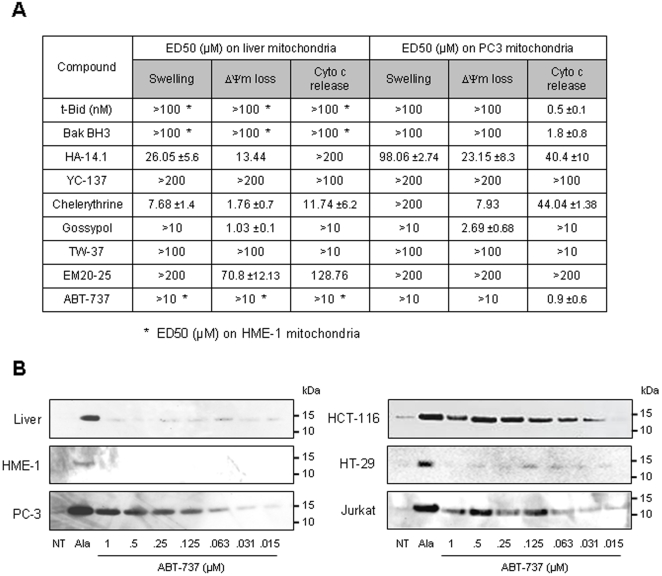
Multiparametric screen of known mitochondria-targeting molecules. **A.** Mitochondria isolated from mouse liver, human non-cancerous (HME-1) and cancerous (PC-3) cells were treated with increasing concentrations of t-Bid, Bak BH3, HA-14.1, YC-137, Chelerythrine, Gossypol, TW-37, EM20-25 and ABT-737 before evaluation of mitochondrial swelling and ΔΨ_m_ loss. Alternatively, mitochondrial supernatant was subjected to ELISA assays for quantification of cytochrome c release. Effective concentration inducing 50% of the maximal effect (EC_50_) is given for swelling (100% of effect with 50 µM Ca^2+^), ΔΨ_m_ loss (100% of effect with 50 µM *m*ClCCP) and cytochrome c release (100% of effect with 20 µg/ml alamethicin) (n = 3 independent experiments). **B.** Mitochondria isolated from mouse liver or HME-1, PC-3, HCT-116, HT-29 and Jurkat cell lines were incubated for 45 min at 30°C with increasing concentrations of ABT-737 and the supernatants were subjected to cytochrome c immunoblot (NT: untreated; Ala: alamethicin 20 µg/ml).

We next evaluated the effect of Bcl-2 inhibitors on mitochondria isolated from mouse liver, human non-cancerous (HME-1) and cancerous (PC-3) cells using 3 parameters: swelling, ΔΨm and cytochrome *c* release (Fig. 2A). The recombinant t-Bid protein induced cytochrome c release (without swelling and ΔΨm loss) from PC-3 mitochondria but had no effect on liver and HME-1 mitochondria at 100 nM. Some BH3 peptides (derived from Bak, Bim, Bax, Bad, Bid, Noxa and Puma) from human or mouse sources were also tested. Among these, only human Bak BH3 and Bim BH3 (Fig. 2A) induced mitochondrio-toxicity to tumor cell (PC-3) mitochondria, while being inactive at 100 µM on liver and HME-1 mitochondria. Noteworthy, even the corresponding mouse BH3 sequences are inactive on mouse liver mitochondria, excluding a misinterpretation due to species specificity (not shown). In contrast to the other small-molecule inhibitors evaluated in this study, only ABT-737 displayed tumor mitochondria specificity, inducing cytochrome c release from PC-3 mitochondria but not from liver and HME-1 mitochondria. The cytochrome c release from PC-3 mitochondria treated with t-Bid and ABT-737 occured without any swelling (Fig. 2A and electronic microscopy, not shown) or ΔΨm loss (Fig. 2A) during a 45 min-treatment, indicating that these conditions occurs a specific OMP. We then extended the study of ABT-737 effects to mitochondria isolated from the HCT-116, HT-29 and Jurkat cancer cell lines (Fig. 2B) and observed different sensitivity to ABT-737. Indeed, HT-29 mitochondria were much less sensitive to ABT-737 than PC-3, HCT-116 and Jurkat, these three laters presenting a similar level of sensitivity to ABT-737. These data suggested that ABT-737 induces cytochrome c release from various but not all mitochondria isolated from cancer cells.

### ABT-737-induced MOMP in cancer cell mitochondria is associated with Bak and/or Bak oligomerization

**Figure 3:**
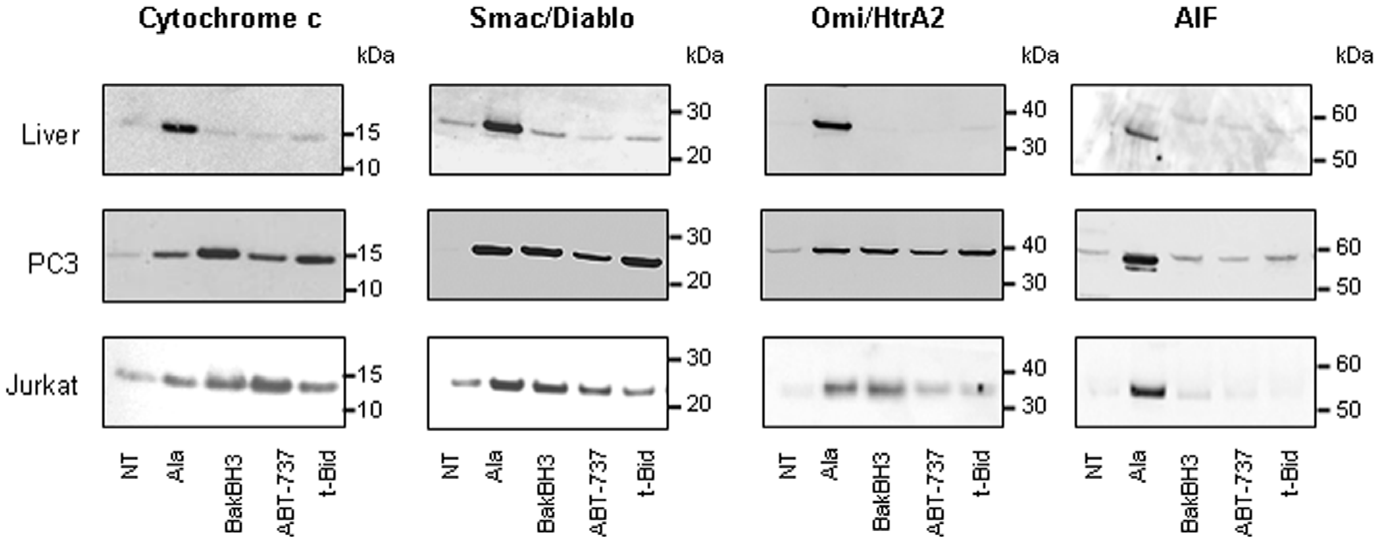
ABT-737 induces relatively large MOMP in cancer cell mitochondria. Isolated mitochondria from mouse liver, PC-3 and Jurkat cells were untreated (NT) or incubated either with alamethicin (Ala; 20 µg/ml; positive control), Bak BH3 peptide (10 µM), ABT-737 (1 µM) or recombinant t-Bid (1 nM) for 45 min. Mitochondrial supernatants were subjected to immunodetection of cytochrome c, Smac/DIABLO, Omi/Htra2 and AIF (Western blots are representative of 3 independent experiments). Note that cytochrome c (15 kDa), Smac/DIABLO (23 kDa), and Omi/Htra2 (37 kDa) but not AIF (56 kDa) are released from cancer cell mitochondria.

**Figure 4:**
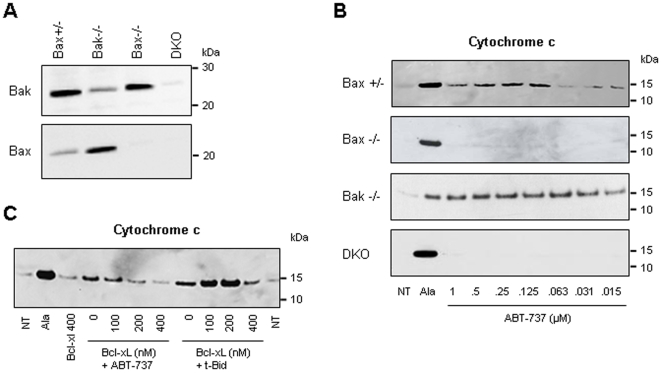
ABT-737 induces a Bax/Bak-dependent cytochrome c release. **A.** Total cell extracts from HCT-116 Bax +/-, Bax -/-, Bak -/- and Bax/Bak -/- (DKO) cell lines were subjected to Bax and Bak immunoblot to control their Bax and Bak content. **B.** Mitochondria isolated from HCT-116 Bax +/-, Bax -/-, Bak -/- and Bax/Bak -/- (DKO) cell lines were incubated with increasing concentrations of ABT-737 and the supernatant was subjected to immunoblot detection of cytochrome c (NT: untreated; Ala: alamethicin 20 µg/ml). **C.** Cytochrome c release induced by t-Bid and ABT-737 is inhibited by an excess of recombinant Bcl-xL. PC-3 mitochondria were incubated with ABT-737 (1 µM) or t-Bid (1 nM) for 45 min after a 5 min-pretreatment with recombinant Bcl-xL (100 to 400 nM) and the supernatant was subjected to anti-cytochrome c immunoblot (NT: untreated; Ala: alamethicin 20 µg/ml). Note that Bcl-xL strongly reduces both t-Bid and ABT-737-induced cytochrome c release (n = 2 independent experiments).

We subsequently investigated if ABT-737-induced OMP was selective to cytochrome c or might allow the release of other apoptogenic mitochondrial factors (Fig. 3). Isolated mouse liver, PC-3 and Jurkat mitochondria were treated with Bak BH3, ABT-737 or t-Bid and the supernatants subjected to immunoblotting. Smac/DIABLO (23 kDa) and Omi/HtrA2 (37 kDa) were released from PC-3 and Jurkat mitochondria whereas AIF (56 kDa) was not (Fig. 3), suggesting that these compounds induced a mitochondria remodeling not sufficient for AIF release. We next used isolated mitochondria from the Bax and/or Bak knock-out HCT-116 cell lines in which absence of Bax and/or Bak was checked by immunoblot (Fig. 4A). We found that ABT-737 induced cytochrome c release from Bax+/- and Bak-/- mitochondria but not from Bax-/- or Bax/Bak double knock-out mitochondria (Fig. 4B). This data pointed out the critical role of Bax in the mechanism of action of ABT-737. Furthermore, t-Bid and ABT-737-induced MOMP was controlled by an excess of Bcl-xL (Fig. 4C) or Bcl-2 (not shown) recombinant proteins, supporting the hypothesis of a formation of a specific channel at the outer membrane [31].

**Figure 5:**
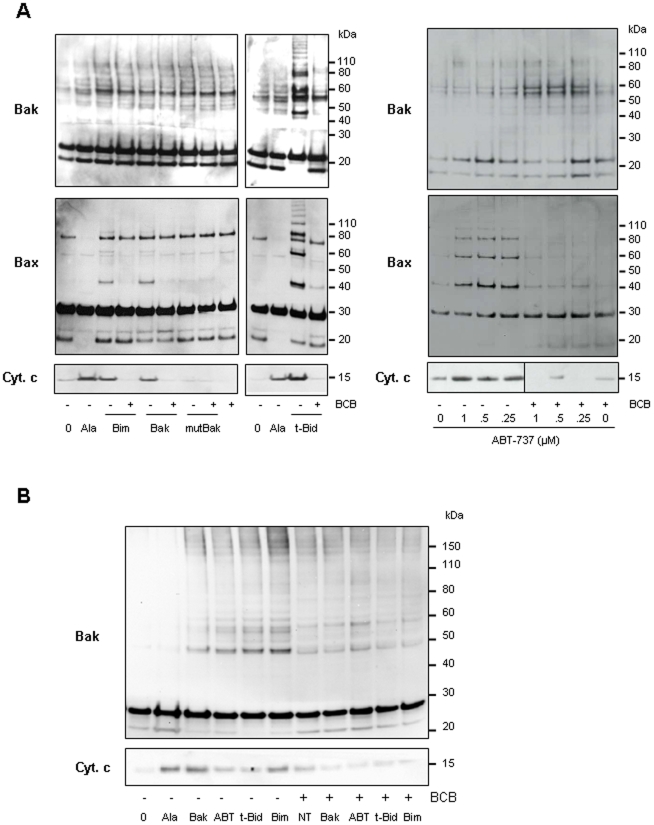
ABT-737 induces Bax and/or Bak oligomerization. Mitochondria isolated from PC-3 (**A**) and Jurkat (**B**) cell lines were incubated or not with the BCB, a Bax Channel Blocker (2 µM (±)-1-(3,6-dibromocarbazol-9-yl)-3-piperazin-1-yl-propan-2-ol) prior to treatment with 1 µM Bim BH3, 10 µM Bak BH3, 10 µM mutated Bak BH3, 1 nM t-Bid or indicated concentrations of ABT-737. Supernatants were analyzed for cytochrome c release (lower panels; NT: untreated; Ala: alamethicin 20 µg/ml) and mitochondrial pellets were treated with the irreversible crosslinker BMH (1 mM). Forty micrograms protein from each reaction was run on SDS-PAGE and immunoblotted with anti-Bax (**A**) or anti-Bak (**A**, **B**) antibodies. Bax/Bak oligomerization accompanies ABT-737-induced cytochrome c release which is inhibited by BCB.

Having found that Bax remained bound to the mitochondrial OM even after a wash with an alkaline homogenization buffer (pH 11.6) (not shown) suggesting an insertion of Bax into the membrane [32], we further wanted to examine if ABT-737 might induce oligomerization of the Bax and Bak pools already associated to tumor cell mitochondria. Similar to t-Bid and Bim or Bak BH3 peptides, ABT-737, induced Bax and/or Bak oligomerization in PC-3 and Jurkat mitochondria, as objectived using the cross-linking agent 1,6-bismaleimidohexane (BMH; Fig. 5). Mutated [L78A; D83A] Bak BH3 peptide was inefficient to induce cytochrome c release and Bax/Bak oligomerization when added to PC-3 mitochondria (Fig. 5A). In PC-3 mitochondria which contain both Bax and Bak, a weak Bak oligomerization occured with BH3 peptides or ABT-737 suggesting a major role for Bax in triggering channels formation in this cell line (Fig. 5A; middle panels). We next used (±)-1-(3,6-dibromocarbazol-9-yl)-3-piperazin-1-yl-propan-2-ol identified by Bombrun and co-workers [33] as a Bax channel blocker (BCB) able to inhibit t-Bid-induced cytochrome c release [33], [34] (Fig. 5A). Pretreatment of cancer cell mitochondria with this BCB prevented cytochrome c release triggered by Bak BH3, Bim BH3, t-Bid or ABT-737 treatment (Fig. 5A). In addition, we found that BCB prevented Bax/Bak oligomerization in response to treatments with ABT-737, as well as t-Bid and Bak or Bim BH3 peptides (Fig. 5A and 5B).

Altogether, these data suggested that ABT-737 triggered the release of apoptogenic proteins from cancer cell mitochondria by formation of multimeric Bax/Bak channels as shown by correlation between Bax and Bak oligomerization and cytochrome c release (Fig. 5).

### ABT-737-induced MOMP in cancer cell mitochondria is associated with particular complex disruptions, depending on the mitochondrial type

**Figure 6:**
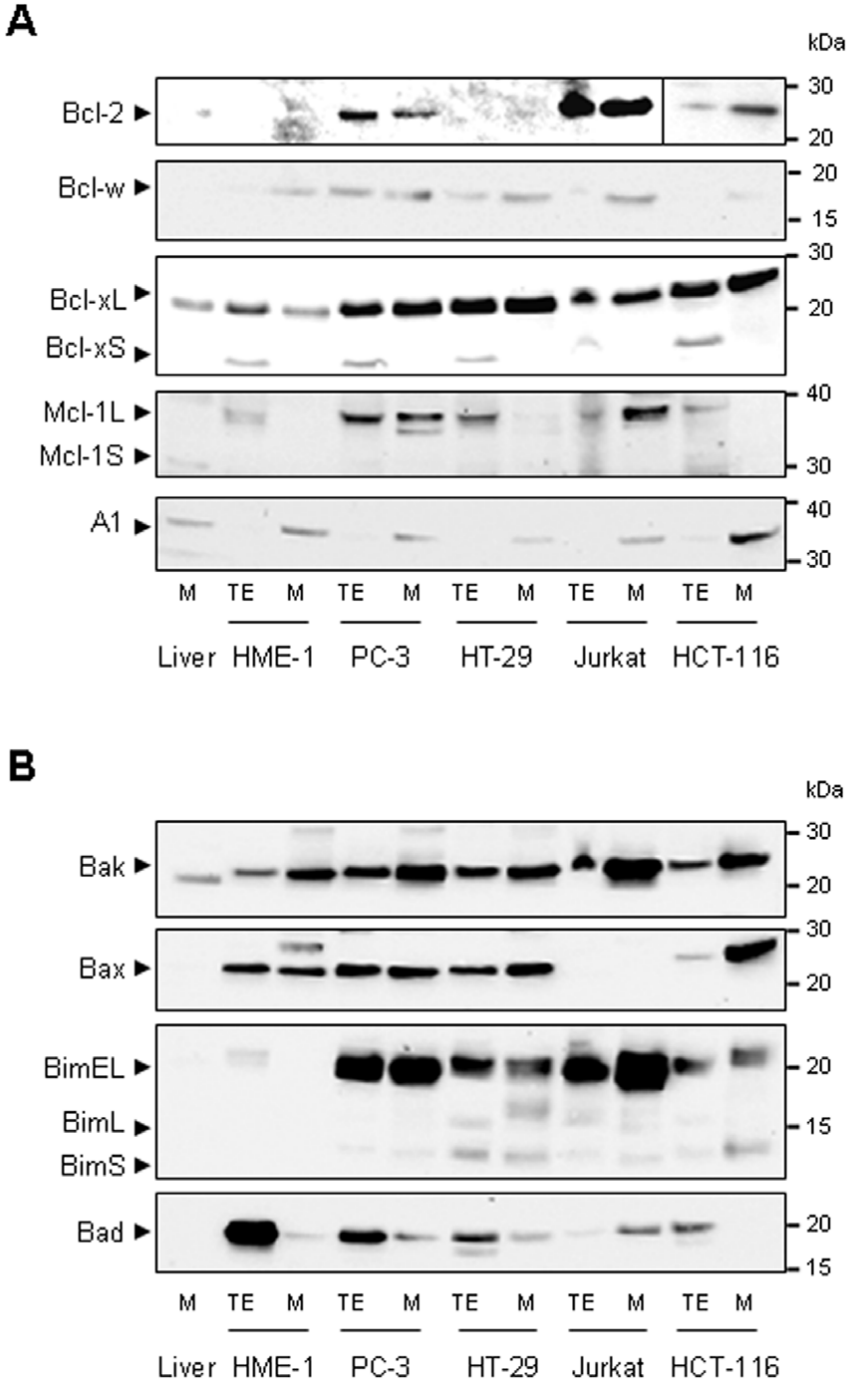
Pro- and anti-apoptotic protein pattern of isolated mitochondria. Total cell extracts (TE) and mitochondrial extracts (M) from PC-3, HT-29, Jurkat and HCT-116 cancer cell lines or from healthy HME-1 cell line and mouse liver were analyzed by Western blot for detection of the anti-apoptotic (**A**) Bcl-2, Bcl-xL, Bcl-w, Mcl-1L and A1 proteins and the pro-apoptotic (**B**) Bak, Bax, Bim, Bad and Mcl-1S proteins.

As differences in sensitivity were observed between the several mitochondrial-types used in this study, we analyzed the pro- and anti-apoptotic Bcl-2 family members associated to the mitochondrial membranes (Fig. 6). Among the anti-apoptotic proteins, Bcl-2 was only present in PC-3, Jurkat and HCT-116 mitochondria, while Bcl-w, Bcl-xL and A1 were detected in all mitochondrial types (Fig. 6A). Interestingly, Bcl-xL was quantitatively more important in cancer cell mitochondria than in their healthy counterpart. Anti-apoptotic Mcl-1L was present in large quantity in PC-3 and Jurkat mitochondria and in smaller quantity in HT-29 mitochondria. Concerning the pro-apoptotic proteins, while Bak was present in all mitochondrial types, Bax was present in PC-3, HT-29, HCT-116 and HME-1 mitochondria but not in Jurkat and liver mitochondria. Among the BH3-only activators, Bim was found in cancer cell mitochondria but not in those from HME-1 and liver (Fig. 6B) while Bid can not be detected in any of these mitochondrial types (not shown). Among the BH3 only sensitizers, Bad was detected at the PC-3, HT-29 and Jurkat mitochondrial membranes (Fig. 6B), while Puma, Noxa, Hrk, Bik, Bok and Bmf were not (not shown). Bcl-2, Bcl-xL and BH3 only sensitizers (ex Bim) might well to be key actors even if it is difficult from such proteomic analysis to explain the differences in sensitivity to ABT-737. Indeed it is noteworthy that HME-1 mitochondria have neither Bim, nor Bcl-2 and only low level of Bcl-xL, which might distinguish them from sensitive cancer cell mitochondria.

**Figure 7:**
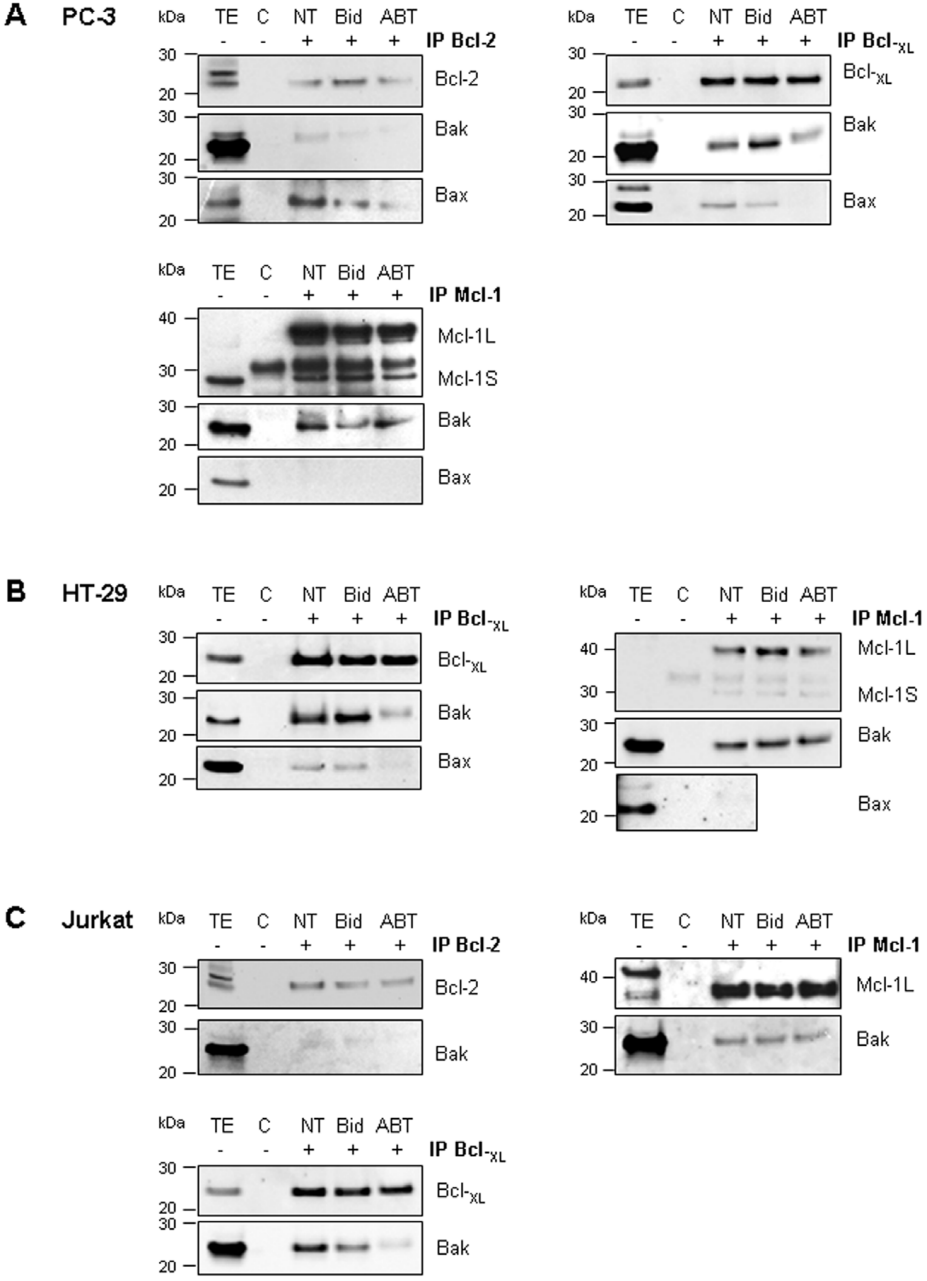
ABT-737 induces Bax and Bak liberation from Bcl-2 and Bcl-xL. Mitochondria isolated from PC-3 (**A**), HT-29 (**B**) and Jurkat (**C**) cells were untreated (NT) or treated with t-Bid (Bid; 2 nM) or ABT-737 (ABT; 1 µM) before to be immunoprecipitated by the antibodies directed against the Bcl-2, Bcl-xL and Mcl-1 anti-apoptotic proteins. Mitochondrial total extracts (TE; positive control of immunoblot; 25 µg) were used as control while a mitochondrial lysate was subjected to immunoprecipitation process without antibody (C; negative control of immunoprecipitation). Thus Western blot analysis was performed to determine bindings between anti-apoptotic proteins and pro-apoptotic Bax and Bak proteins (representative Western Blots of 3 independent experiments).

**Figure 8:**
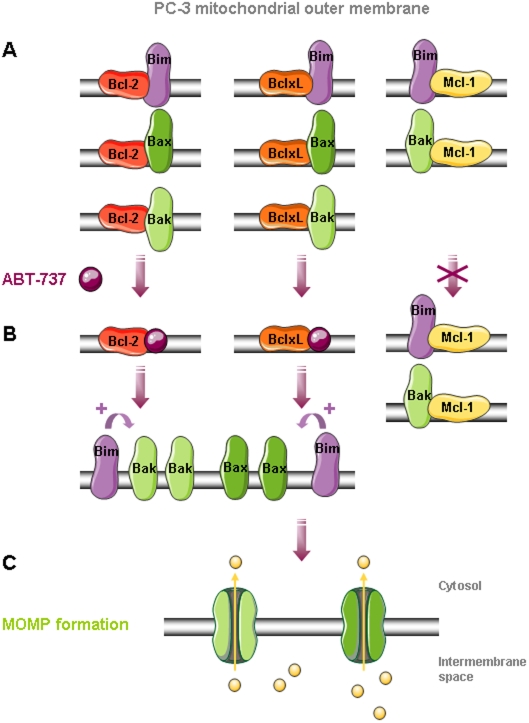
MOMP formation in PC-3 mitochondria in response to ABT-737. **A.** Bax, Bak and Bim are sequestred by Bcl-2, Bcl-xL and Mcl-1 at the outer mitochondrial membrane. **B.** In response to ABT-737, Bax, Bak and Bim proteins are liberated from Bcl-2 and Bcl-xL but not from Mcl-1L. Thus Bim can directly enhance Bax and Bak oligomerization triggering MOMP formation (**C**) and release of pro-apoptotic proteins such as cytochrome c in the cytosol.

As ABT-737 is acting by complex disruption between pro- and anti-apoptotic proteins, we next investigated some complex disruptions by co-immunoprecipitation in PC-3, HT-29 and Jurkat mitochondria treated with ABT-737 (Fig. 7). Whatever the cell line we detected similar bindings: Bcl-xL to Bax and Bak, Bcl-2 to Bax and weakly to Bak, Mcl-1 only to Bak (Fig. 7) and Bcl-w to Bax (not shown). We observed that ABT-737-induced cytochrome c release is correlated with Bax, Bak (Fig. 7) and Bim (not shown) liberation from Bcl-xL and Bcl-2. However, ABT-737 had no effect on Bak and Bim sequestration by Mcl-1 (Fig. 7), or Bax sequestration by Bcl-w (not shown), these complexes remaining after treatment. These results suggested that Bax, Bak and Bim liberation from Bcl-2 and Bcl-xL in response to ABT-737 was responsible for channels formation and cytochrome c release in PC-3 (Fig. 8) and Jurkat mitochondria. In contrast, HT-29 mitochondria containing less Bim and being deprived of Bcl-2 were less sensitive to ABT-737 treatment, suggesting a major role for Bcl-2 and Bim in ABT-737 sensitivity.

## Discussion

In this study, we used high quality controlled isolated mitochondria to compare the effects of putative Bcl-2 inhibitors and try to explore the mechanism of action of ABT-737. We used five different parameters to evaluate their integrity and functionnality: cytochrome c oxidase accessibility to exogenous cytochrome c (not shown), respiratory control values, capacity for matrix swelling, transmembrane potential values and release of apoptogenic factors like cytochrome c (OMP) (Fig. 1). Comparision of compounds-effect on each mitochondrial type requires similarly high levels of purity and intactness of mitochondrial preparations whatever their sources (cultured cells or healthy tissue). This was solved by large-scale cell cultures and purification of mitochondria by differential centrifugations plus Percoll density gradient. Using this method, both isolated mouse liver and cancer cell mitochondria present similar quality and response to calcium (Fig. 1).

Surprisingly enough most compounds identified as Bcl-2 inhibitors were found to act on healthy mitochondria at least on one integrity parameter. For instance, we observed that HA-14.1, Chelerythrine, Gossypol and EM20-25 induced MMP in mouse liver mitochondria, while other Bcl-2 family inhibitors were found to be inactive (YC-137 and TW-37). Appart from t-Bid, Bak BH3, Bim BH3 which are from proteic origins, only ABT-737 demonstrated selective tumor mitochondrio-targeting indicated by OMP and release of pro-apoptotic factors (Fig. 2). Previous observations have proven that ABT-737 can induce OMP either when mitochondria originate from cells “primed” by death signals (for instance in IL-3-deprived lymphocytes [28], or in TNF-pulsed HeLa cells [35]), or when isolated mitochondria are co-treated with BH3 peptide (for instance with Noxa BH3 on MEF mitochondria [13]). For the first time, we demonstrated that ABT-737 can itself induce OMP on mitochondria isolated from unprimed tumor cell lines. Concerning t-Bid, our isolated liver and HME-1 healthy mitochondria were not sensitive to the recombinant protein t-Bid. This absence of effect on liver mitochondria could be explained by the high purity and stability of our mitochondrial preparations. Bcl-2 family proteins detected on both normal and cancer cells mitochondria (Fig. 6) remind present after alcaline washes (not shown) indicating that they are not associated by electrostatic interaction with the mitochondrial membranes and are not coming from residual cytosol or endoplasmic reticulum.

The recombinant t-Bid protein, Bak BH3, Bim BH3 and ABT-737 triggered a release of apoptogenic proteins from PC-3 and Jurkat mitochondria by formation of channels large enough to release proteins such as Omi/HtrA2 (37 kDa) (Fig. 3). OMP appears independent on PTP since it is not inhibited by known PTP inhibitors like ADP, cyclosporin A and bongkrekic acid (not shown). The absence of mitochondrial membrane alterations (no swelling and ΔΨm loss) (Fig. 2A) and the release of the smallest apoptotic factors under treatment (Fig. 3) suggested that ABT-737 induced the formation of a specific channel and not a mitochondrial membrane rupture, similarly to the Bax[53–86] BH3 peptide in Polster *et al.* [36]. Accordingly, discriminative release of apoptogenic factors has already been shown in isolated HeLa mitochondria treated with t-Bid [37]. This finding is compatible with the previous description of an apoptosome-dependent loop where downstream caspases need to be activated to trigger mitochondrial release of AIF and EndoG, secondary to the release of cytochrome c, Omi/HtrA2 and Smac/DIABLO [37]. In cellular model, ΔΨm loss and cytochrome c release were simultaneously detected in response to ABT-737 [38], [39] contrary to what was observed with our conditions in cell-free system. Our screening method seems to be a real time process that allows detection of direct and early effects of compounds on mitochondria, without interferences induced by cytosolic compartment.

We have also shown that (1) HCT-116 Bak-/-, but not Bax-/-, mitochondria are sensitive to ABT-737 (Fig. 4A), (2) ABT-737-induced cytochrome c release on PC-3 mitochondria are controlled by an excess of Bcl-xL (Fig. 4C) and (3) inhibition of Bax and Bak oligomerization by BCB is sufficient to block cytochrome c release (Fig. 5A and B). These findings indicate that equilibrium between pro-apoptotic and anti-apoptotic members of the Bcl-2 family plays an essential role in the ABT-737 mechanism of action.

We have thus demonstrated that Bax and Bak oligomerization at the PC-3 mitochondrial membrane is induced by Bak and Bim BH3 peptides, t-Bid or ABT-737 treatments (Fig. 5A), Bax and Bak both being inserted as a monomeric form in untreated normal (HME-1) and tumoral (PC-3) cell mitochondria. However, numerous studies have been performed showing Bax oligomerization and subsequent membrane insertion using recombinant Bax and isolated mitochondria or liposomes [40]–[42]. These studies have led to opposite conclusions on the kinetic of Bax pores activation. However, more recently, it has been shown that oligomerization of Bax occurs at the mitochondrial level rather than in the cytosol [43]–[45]. Thus, using c-myc null cells, Annis and co-workers showed that Bax-induced mitochondrial permeabilization results from oligomerization of transmembrane monomers rather than insertion as preformed oligomers [43].

Some Bcl-2 family proteins, such as the BH3 only activator Bim or the anti-apoptotic proteins Bcl-2 and Mcl-1L are especially present at cancer cell mitochondria. In contrast with previous observations [28], [29], [46], Mcl-1L expression at the mitochondria was not sufficient in our hands to prevent MOMP formation in response to ABT-737. For instance, PC-3 and Jurkat mitochondria are sensitive to low concentrations of ABT-737 despite a high Mcl-1L content (Figs 2 and 6), while HT-29 mitochondria with low level of Mcl-1L are relatively resistant to ABT-737. We show here that at the molecular level, ABT-737 allows pro-apoptotic proteins Bcl-2 and Bcl-xL but neither Mcl-1L nor Bcl-w to liberate Bax, Bak and Bim (Figs 7 and 8). Bim, as activator of Bax and Bak oligomers, plays a key role in ABT-737-induced apoptosis [47]. This suggests that sensitivity to ABT-737 depends on Bim presence and on the balance between the quantity of Bcl-2 and Bcl-xL *versus* Mcl-1L and Bcl-w, explaining resistance of some mitochondrial types, deprived of Bcl-2 (HT-29 mitochondria) or both Bcl-2 and Bim (HME-1 and liver mitochondria). Interestingly, HME-1 mitochondria are less sensitive to t-Bid than cancer cell mitochondria despite the presence of Bax and Bak (Fig. 6). This observation suggests a slight difference in Bax and Bak regulation in healthy and cancer mitochondria isolated from cultured cell lines. Extended investigations are needed to explain this difference.

Finally, the comparative approach based on isolated “pathological” *versus* “healthy” mitochondria appears to be a usefull tool to identify Bcl-2 inhibitors and investigate their mechanism of action on a particular cell type. It also represents a reliable, fast, and predictive screening tool, tailored for selecting series or compounds with selective toxicity profile against mitochondria from cancer cell lines and devoid of toxicity against healthy mitochondria.

## Materials and Methods

### Peptides and reagents

The human Bak BH3 (CMGQVGRQLAIIGDDINRRYDS), mutated [L78A; D83A] Bak BH3 and Bim BH3 (CEIWIAQELRRIGDEFNAYYAR) peptides were purchased at Abgent (Interchim SA, Montluçon, France). The low molecular weight mitochondrio-toxic compounds used are: HA-14.1, YC-137, Chelerythrine, EM20-25 and Gossypol (Sigma-Aldrich, St Quentin Fallavier, France); recombinant t-Bid (gift from Dr. J.C. Martinou); TW-37 and ABT-737 (synthetized by Almac Sciences, UK). Other compounds used are: Bax channel blocker (BCB; (±)-1-(3,6-dibromocarbazol-9-yl)-3-piperazin-1-yl-propan-2-ol; Calbiochem, San Diego, CA); cyclosporin A (CsA; BIOMOL Research Laboratories, Inc, Tebu Bio SA, Le Perray en Yvelines, France); oligomycin and mClCCP (*m*-chlorocarbonylcyanide phenylhydrazone, Sigma Aldrich) and the recombinant protein Bcl-xL (Oncogene™ Research products, Merck, VWR international, Fontenay sous bois, France).

### Purification of mice liver and tumor cell lines mitochondria

Liver mitochondria were isolated from 6 weeks old BALB/cByf female mice (Charles River, Saint Germain sur L'arbresle, France) as previously described [30]. Animal housing, care and application of experimental procedures were conducted in compliance with the European Community guidelines for the care and use of experimental animals (Animal Health Regulations, Council Directive No. 86/609/EEC of 24^th^ November 1986). The experimental procedure on mice was rewieved and approved by the Bichat - Debré Hospitals Ethics Committee. Purified organelles were re-suspended in homogeneization buffer (300 mM sucrose, 5 mM TES pH 7.2, 0.2 mM EGTA, 1 mg/ml BSA). Mitochondria were also isolated from human mammary gland epithelial cells immortalized by stable expression of the human telomerase reverse transcriptase [48] (hTERT-HME-1; ATCC) and human cancer cell lines (PC-3, prostate adenocarcinoma (NCI); HT-29, colon adenocarcinoma (ATCC); Jurkat, acute T cell leukemia (ATCC); HCT-116, colon adenocarcinoma, deficient or not for Bax and/or Bak (from Dr. Peter Daniel and Prof. Bert Vogelstein). Briefly, adherent cells were harvested with Trypsin/EDTA, centrifuged at 750 rpm for 10 min, washed in buffer A (100 mM sucrose, 1 mM EGTA, 20 mM MOPS, pH 7.4 and 1 mg/ml BSA) before cell break with a Dounce homogenizer. The suspension was centrifuged twice at 2 500 g for 5 min and the resulting supernatant at 10 000 g for 10 min at 4°C. The pellet was resuspended in buffer B (300 mM sucrose, 1 mM EGTA, 20 mM MOPS pH 7.4, 1 mg/ml BSA and 1 mM PMSF) and the homogenate was layered on a two phase percoll density gradient. After centrifugation, mitochondria (layered at the interface) were removed, washed with homogeneization buffer for 10 min. at 10 000 g, and resuspended in homogeneization buffer. To ensure quality of mitochondrial preparations, samples were subjected to various assays for integrity and functionnality including cytochrome oxydase accessibility, respirometry and FSC/SSC FACScan (BD Bioscience, Germany) analysis in the presence or absence of Mitotracker™ green (ΔΨm insensitive) and Mitotracker™ red (ΔΨm sensitive) as described in [30].

### Detection of large amplitude swelling and ΔΨ_m_

Our screening platform is dedicated to the real-time co-monitoring of mitochondrial swelling and ΔΨ_m_. Freshly isolated mitochondria are distributed in 96-well plates in buffer D (200 mM sucrose, 5 mM succinate, 10 mM MOPS pH 7.4, 1 mM H_3_PO_4_, 2 µM rotenone and 10 µM EGTA) supplemented with 1 µM rhodamine 123 (Rh123; Molecular Probes™, Invitrogen, Cergy Pontoise, France) followed by the addition of serial dilutions of small compounds or synthetic peptides. Absorbance at 545 nm and Rh123 fluorescence (excitation 485 nm, emission 535 nm) are recorded during 30 cycles of 1 min using a fluorescence multi-well plate reader (Infinite, Tecan®, Männedorf, Switzerland). CaCl_2_ (50 µM) and mCICCP (20 µM) treatments were considered as the 100% baseline for the swelling and ΔΨ_m_ loss, respectively. The EC_50_ are the concentrations corresponding to 50% of maximal swelling and 50% of maximal ΔΨ_m_ loss at 30 min.

### Determination of cytochrome c, Smac/DIABLO, Omi/Htra2 and AIF release

Isolated mitochondria (20 µg proteins) were incubated with 20 µg/ml Alamethicin (Ala, positive control, 100% baseline for ELISA), small molecules or synthetic compounds in buffer D for 30 or 45 min at 30°C. After a 7 min centrifugation at 10 000 g, proteins contained in supernatant were analyzed for quantification of cytochrome c release using ELISA kits from MBL (Cliniscience, Montrouge, France) for liver mitochondria and from Biosources (Invitrogen) for tumor cell lines mitochondria and/or runned on NuPAGE® 4–12% Bis-Tris gels (Invitrogen) and transfered to nitrocellulose using the iBlot™ Dry Blotting System (Invitrogen). Subsequently the membrane was blocked for 1 h with 5% low fat milk in TBS-0.1% tween-20 (TBST) and incubated with anti-cytochrome c mouse monoclonal IgG_2b_ antibody (BD Bioscience Pharmingen; 1/500); or anti-Smac/DIABLO (Calbiochem; 1/3000), anti-Omi/HtrA2 (R&D Systems, Lille, France; 1/2000), anti-AIF (Upstate Biotechnology, Millipore, Billerica, MA;1/1000) rabbit polyclonal IgG antibodies. After 1 h-washes with TBST, the membrane was treated with horseradish peroxidase-coupled anti-mouse IgG (Promega, Charbonnière, France; 1/2500) or anti-rabbit IgG (Amersham Biosciences, Piscataway, NJ; 1/3000) for ECL detection (Amersham Biosciences).

### BMH cross-linking

A 20 mM stock of 1,6-bismaleimidohexane (BMH; Pierce) prepared in DMSO was added to treated mitochondria at a 1∶20 dilution in buffer D. After 1 hour at 30°C, mitochondria were centrifuged 10 min at 12 000 g and dissolved in 4x NuPAGE sample buffer (Invitrogen) with DTT (dithiothreitol) before SDS-PAGE electrophoresis.

### Determination of mitochondrial membrane-associated proteins

Proteins were analyzed by Western blot using: anti-Bcl-2 (C-2, Santa Cruz Biotechnology, Tebu Bio; 1/500) mouse monoclonal IgG_1_ antibody; anti-Bcl-w (31H4, Cell Signalling; 1/1000), anti-Mcl-1 (Y37, Abcam; 1/1000), anti-A1 (BioVision, Cliniscience; 1/400), anti-Bax (N20; Santa Cruz Biotechnology; 1/1000), anti-Bak (Upstate Biotechnology; 1/1000), anti-Bcl-xL (Pharmingen BD Bioscience; 1/1000), anti-Bim (Calbiochem; 1/1000) or anti-Bad (Cell Signaling, Ozyme, Montigny le Bretonneux, France; 1/1000) rabbit polyclonal IgG antibodies.

### Immunoprecipitation experiments

Isolated mitochondria (600 µg) were incubated or not with t-Bid (2 nM) or ABT-737 (1 µM) during 40 min at 30°C. After centrifugation for 10 min at 10 000 g, mitochondria were lysed by incubation at 4°C during 15 min in 1 ml of CHAPS buffer (Hepes 50 mM, KCl 150 mM, EDTA 1 mM, EGTA 1 mM, CHAPS 0.2%, NP40 0.05%, NaF 5 mM and PMSF 1 mM). After centrifugation for 10 min at 10 000 g, supernatant was incubated for 16 h at 4°C with protein G agarose (Pierce) and antibodies: mouse anti-Bcl-2 (Santa Cruz clone C-2), anti-Mcl-1 (Santa Cruz clone 22) or rabbit anti-Bcl-xL (Cell Signaling). The precipitate was washed 4 times with CHAPS buffer and boiled 5 min in loading buffer before immunoblot analysis.

### Electron microscopy

Isolated mitochondria were fixed with 2% glutaraldehyde in 0.1 M Na-cacodylate buffer, pH 7.2 for 3 h at 4°C. After 2 washes with 0.2 M sucrose in 0.1 M Na-cacodylate buffer, pH 7.2, the specimens were then postfixed with 1% osmium tetroxide containing 1.5% potassium cyanoferrate, dehydrated in gradual ethanol (30–100%) and embedded in Epon. 70 nm thin sections were collected onto 200 mesh cupper grids, counterstained with uranyl acetate and lead citrate before examination with a Zeiss EM 902 transmission electron microscope at 80 Kvolt (MIMA2, Electron Microscopy Platform-GPL, Jouy-en-Josas). Microphotographies were acquired using MegaView III CCD camera and analysed with ITEM software (Eloïse SARL, Roissy, France).

### Polarographic studies

Isolated mitochondria were incubated in a magnetically stirred 1.5 ml cell with a Clark type oxygen electrode (Hansatech Instruments Ltd, Norfolk, UK) thermostated at 37°C, in 500 µl of a medium consisting of 0.3 M mannitol, 10 mM phosphate buffer (pH 7.3), 10 mM KCl, 5 mM MgCl_2_ and 1 mg/ml BSA as previously described [49]. ADP addition causes a sudden burst of oxygen uptake when the ADP is converted into ATP characterized by an actively respiring state (state 3) respiration followed by a slower rate after all the ADP has been phosphorylated to form ATP (state 4). The ratio [state 3 rate] / [state 4 rate] which is called the respiratory control index (RCI) indicates the tightness of the oxidative phosphorylation, thus referring to the respiratory chain functionality and the quality of the mitochondrial preparation. The inhibitor Oligomycin A which blocks respiration in coupled mitochondria and the uncoupling agent mClCCP were added in the case of tumoral cell mitochondria.
